# The effect of prone versus supine positioning in mortality among adult patients with moderate to severe acute respiratory distress syndrome: a meta-analysis

**DOI:** 10.1186/2197-425X-3-S1-A93

**Published:** 2015-10-01

**Authors:** J Tabula, LS Ordillo, PP Remalante, A Wang

**Affiliations:** Department of Medicine, University of the Philippines Manila - Philippine General Hospital, Manila, Philippines

## Introduction

Prone positioning has been proven to increase oxygenation among patients with ARDS. However, individual RCTs did not show a significant mortality reduction among ARDS patients in prone compared to supine positioning except in the recent PROSEVA study.

## Objectives

This meta-analysis assessed the effectiveness of prone versus supine positioning among patients with ARDS in reducing 28-day, 60-day, 90-day, and ICU mortality. Incidence of adverse events (ventilator-associated pneumonia, pneumothorax, endotracheal tube obstruction and unplanned endotracheal tube extubation/displacement) and length of ICU stay were also be evaluated.

## Methods

We used literature search of MEDLINE, Current Contents, Cochrane Central Register of Controlled Trials, Google Scholar, Science Direct, and HighWire Press to identify relevant RCTs for our meta-analysis. We used the following search strategy and MeSH/key terms: (prone OR prone ventilation) AND (ARDS OR acute respiratory distress syndrome). We limited our search to clinical trials. No limits are set for language of publication. We performed manual search of the references from original studies and review articles to identify any additional relevant trials and increase completeness. We searched for electronic conference proceedings of the American Thoracic Society and unpublished and ongoing trials in ClinicalTrials.gov and Current Controlled Trials. Odds ratio with 95% CI was computed for dichotomous outcomes using Mantel-Haenszel and random-effects model.

## Results

Four RCTs met our inclusion criteria, including 502 patients randomized to prone ventilation and 482 patients to supine ventilation. All studies had low risk of bias. Overall, prone ventilation did not reduce 28-day (OR = 0.64; 95% CI = 0.39-1.03; p = 0.05; *I*^2^ = 62%), 60-day (OR = 0.73; 95% CI = 0.44-1.23; p = 0.02; *I*^2^ = 66%), and 90-day (OR = 0.71; 95% CI = 0.39-1.27; p = 0.03; *I*^2^ = 73%) except for ICU mortality (OR = 0.59; 95% CI = 0.38-0.92; p = 0.09; *I*^2^ = 58%). Length of ICU stay was not reduced (OR = 1.28; 95% CI = -2.10-4.66; p = 0.34; *I*^2^ = 13%). Prone ventilation did not increase adverse events except for endotracheal tube obstruction (OR = 2.02; 95% CI = 1.35-3.03; p = 0.88; *I*^2^ = 0%).

## Conclusions

Our meta-analysis does not suggest a beneficiary effect of prone positioning on mortality (28-day, 60-day, 90-day) except for ICU mortality among overall moderate to severe ARDS patients. In addition, our data suggests that ventilation in the prone position increases the incidence of airway obstruction but does not shorten days of ICU stay nor increase incidence of ventilator-associated pneumonia, unplanned endotracheal tube extubation/displacement, or pneumothorax.

